# Gut microbiome and intestinal inflammation in preclinical stages of rheumatoid arthritis

**DOI:** 10.1136/rmdopen-2023-003589

**Published:** 2024-01-30

**Authors:** Benoît Thomas P Gilbert, Raul Yhossef Tito Tadeo, Celine Lamacchia, Olivia Studer, Delphine Courvoisier, Jeroen Raes, Axel Finckh

**Affiliations:** 1 Division of Rheumatology, HUG, Geneva, Switzerland; 2 Geneva Centre for Inflammation Research, UNIGE, Geneva, Switzerland; 3 KU Leuven, Department of Microbiology, Immunology, and Transplantation, Rega Institute for Medical Research, B-3000 Leuven, Belgium; 4 VIB, Center for Microbiology, B-3000 Leuven, Belgium

**Keywords:** Rheumatoid Arthritis, Autoimmune Diseases, Autoimmunity, Permeability, Rheumatoid Factor

## Abstract

**Background:**

Faecal *Prevotellaceae*, and other microbes, have been associated with rheumatoid arthritis (RA) and preclinical RA. We have performed a quantitative microbiome profiling study in preclinical stages of RA.

**Methods:**

First-degree relatives of patients with RA (RA-FDRs) from the SCREEN-RA cohort were categorised into four groups: controls, healthy asymptomatic RA-FDRs; high genetic risk, asymptomatic RA-FDRs with two copies of the shared epitope; autoimmunity, asymptomatic RA-FDRs with RA-associated autoimmunity; and symptomatic, clinically suspect arthralgias or untreated new-onset RA.

Faecal samples were collected and frozen. 16S sequencing was performed, processed with DADA2 pipeline and Silva database. Cell counts (cytometry) and faecal calprotectin (enzyme-linked immunosorbent assay, ELISA) were also obtained. Microbial community analyses were conducted using non-parametric tests, such as permutational multivariate analysis of variance (PERMANOVA), Wilcoxon and Kruskal-Wallis, or Aldex2.

**Results:**

A total of 371 individuals were included and categorised according to their preclinical stage of the disease. Groups had similar age, gender and body mass index. We found no significant differences in the quantitative microbiome profiles by preclinical stages (PERMANOVA, R2=0.00798, p=0.56) and, in particular, no group differences in *Prevotellaceae* abundance. Results were similar when using relative microbiome profiling data (PERMANOVA, R2=0.0073, p=0.83) or Aldex2 on 16S sequence counts. Regarding faecal calprotectin, we found no differences between groups (p=0.3).

**Conclusions:**

We could not identify microbiome profiles associated with preclinical stages of RA. Only in a subgroup of individuals with the most pronounced phenotypes did we modestly retrieve the previously reported associations.

**WHAT IS ALREADY KNOWN ON THIS TOPIC**

- Faecal microbes, such as *Prevotellaceae*, have been associated with rheumatoid arthritis (RA) and preclinical RA.
- Intestinal inflammation and permeability modulate arthritis severity in mice models.

**WHAT THIS STUDY ADDS**

- Unexpectedly, we found no association between faecal microbes and preclinical stages of RA.
- Faecal calprotectin, a proxy or intestinal inflammation, was mostly normal in this at-risk population.

**HOW THIS STUDY MIGHT AFFECT RESEARCH, PRACTICE OR POLICY**

- This study questions the transposability of microbiome studies derived from animal models of arthritis to humans when considering the onset of autoimmunity in individuals at higher risk for RA.

## Introduction

Rheumatoid arthritis (RA) is a rheumatic autoimmune disease affecting about 0.5% of the population.^1^ It results from a multistep process whereby environmental risk factors induce autoimmunity in genetically susceptible individuals.^2^ RA onset is insidious, going through asymptomatic or paucisymptomatic phases that are called ‘preclinical’ stages of RA.^3^ Among the risk factors driving the transition towards RA development, mucosal health has recently gained much attention.^4^ Overall, the ‘mucosal origin hypothesis’ postulates that chronic mucosal inflammation (gut, oral cavity and lungs) drives the initial loss of immune tolerance for self-structures. Mucosal microbiomes, periodontitis, smoking and other environmental factors would also be involved in the pathogenesis by promoting or aggravating local mucosal inflammation.^4^

Drawing a parallel between reactive arthritis and RA, investigators in the mid-20th century were already curious about stool microbiome of patients with RA as it is the largest human reservoir of foreign antigens and microbes.^5–7^ But it is only in the 2000s that genomic probing and sequencing techniques popularised microbiome profiling.^8^ The first 16S ribosomal RNA (rRNA) gene analyses (using fluorescent probes) found a quantitative *decrease* of *Bacteroides*, *Prevotella* and *Porphyromonas* genera in RA patients compared with control patients.^9 10^ Then, between 2013 and 2019, three studies using 16S rRNA sequencing demonstrated an increased relative abundance of *Prevotellaceae*, particularly *Prevotella copri (P. copri)*, in patients with preclinical RA and early stages of RA when compared with healthy controls.^11–13^ However, subsequent studies contradicted the aforementioned findings.^14–20^ Moreover, the variability in the population’s characteristics adds complexity to the interpretation of these correlations. Microbiome profiling can also be accomplished through ‘shotgun’ sequencing, which aims at sequencing all the available DNA.^21^ Shotgun sequencing studies confirmed differences in the composition of the gut microbiota of patients with RA compared with control groups. Still, perplexingly, the taxa implicated in these differences do not consistently align with those reported by 16S sequencing studies.^22–24^

To clarify the matter, mouse models of RA have been colonised with bacteria derived from faeces of patients with RA. Mice-derived evidence revealed how certain ‘arthritogenic’ bacteria could exacerbate intestinal inflammation and arthritis.^11 13 14 25–27^ For instance, in 2016, Maeda *et al* demonstrated that germ-free mice colonised with *Prevotella*-dominated microbiota from patients with RA had an increased number of intestinal T helper (Th) 17 cells and developed severe arthritis when treated with zymosan, compared with ‘healthy control microbiota’ colonisation.^13^ Other microbes have likewise proven to aggravate arthritis in mice models; they include *Eggerthella*,^14 22 28^ *Collinsella*,^14 16 28^ *Subdoligranulum*^27^ or *Fusobacterium nucleatum (F. nucleatum)*.^19^ The involved mechanisms may sometimes differ, as the *Subdoligranulum* strain seemed to stimulate Th17 cell expansion and B cell activation in gut lymphoid follicles,^27^ while *F. nucleatum* rather promoted arthritis by secreting antigenic outer membrane vesicles able to translocate in joints and trigger inflammation.^19^ Nevertheless, the presence of these ‘arthritogenic’ microbes in humans, during the initiation of autoimmunity in preclinical stages of RA, remains uncertain. Additionally, it is unclear whether these microbes are associated with subclinical intestinal inflammation in the preclinical RA population.

Our study is an attempt to expand on previous findings using a quantitative methodology. Specifically, we focused on the presence of *Prevotellaceae* bacteria in an untreated population at various preclinical stages of RA.^12^ We also assessed intestinal inflammation using faecal calprotectin, which is an antimicrobial protein translocated into the extracellular fluids, or the intestinal lumen, by activated neutrophils.^29^

## Methods

### Study population

The SCREEN-RA cohort has been extensively described elsewhere.^30^ Briefly, since 2009, the SCREEN-RA cohort has recruited more than 1500 RA-FDRs of established patients with RA, across Switzerland. After having provided a baseline serum sample, participants are followed up yearly using online questionnaires. Individuals at higher risk of developing RA, presenting with autoantibodies associated with RA or clinically suspect arthralgia (CSA), are monitored more closely and reinvited for further study visits on a yearly basis until the development of RA. Participants were excluded if they developed another autoimmune disease or if they initiated an immunosuppressive treatment.

In parallel, untreated patients with new-onset RA from the Geneva rheumatology division were also invited to participate in the study as positive controls, before initiating disease-modifying anti-rheumatic drug (DMARD) therapy or glucocorticoids.

### Study design

This study is nested within the SCREEN-RA cohort study. We performed a cross-sectional comparison between four distinct at-risk groups, defined based on the current recommendations (details below).^3^ We purposely did not recruit healthy controls from the general population, in order to minimise by design possible confounding factors such as habits, economic status and genetic background. Furthermore, we excluded treated patients with RA as DMARDs potentially modify the gut microbiome.^31^

### Sample collection

Between September 2019 and October 2021, SCREEN-RA participants have been invited to provide a stool sample paired with a serum sample. Participants were provided with stool collection devices allowing the creation of several aliquots of stool and proceeded to stool sampling at home. They temporarily froze the fresh stool sample at −20°C and rapidly brought it to the study centres to be stored at −80°C without any additive, as previously described.^32^ During the study visit, a blood sample was also taken, clotted and centrifuged to store several serum aliquots at −80°C according to SCREEN-RA standard operating procedures.^30^ The average time difference between stool sampling and serum sampling was 2.55 days (SD = 13.6 days).

### Serum samples processing

Each serum sample was assessed for rheumatoid factor (RF) and anti-citrullinated peptide antibodies (ACPA). ACPA serology was defined as positive if at least one of the following tests was positive: CCPlus Immunoscan (anti-CCP2) IgG ELISA (Svar Life Science, Malmö, Sweden), QUANTA Lite CCP3.1 IgG/IgA ELISA (INOVA Diagnostics), QUANTA Lite CCP3 IgG ELISA (INOVA Diagnostics) or QUANTA Flash CCP3 IgG CIA (INOVA Diagnostics). Similarly, RF was defined as positive if at least one of the following tests was positive: QUANTA Lite RF IgM ELISA (INOVA Diagnostics), QUANTA Lite RF IgA ELISA (INOVA Diagnostics), Elia RF IgM (Phadia AB) or Elia RF IgA (Phadia AB). Results were recorded in the database for each test and interpreted based on the manufacturer’s recommended cutoffs.

### Stool sample processing

DNA was extracted from a thawed stool aliquot (~200mg) using Qiagen MagAttract PowerMicrobiome DNA/RNA Kit bead-beating kit on a robotised platform. Briefly, the manufacturer’s protocol was modified by the addition of a heating step at 90°C for 10 min after vortexing and by the exclusion of the steps where DNA is removed. DNA samples were then randomised on 96 well plates, and for bacterial and archaeal characterisation, extracted DNA (dilution 1:10) was further amplified in triplicate using 16S rRNA primers 515F (5’-GTGYCAGCMGCCGCGGTAA-3’) and 806R (5’-GGACTACNVGGGTWTCTAAT-3’) targeting the V4 region, modified to contain a barcode sequence between each primer and the Illumina adaptor sequences to produce dual-barcoded libraries, as previously done.^33^ Deep sequencing was performed on a MiSeq platform (2×250 paired-end reads, Illumina).

Microbial loads of stool samples were measured as described previously.^34^ Moisture content was determined as the percentage of mass loss after lyophilisation from 0.2 g frozen aliquots of non-homogenised faecal material (−80°C) as previously described.^34^ Finally, faecal calprotectin concentrations were determined using the fCAL enzyme-linked immunosorbent assay (ELISA) Kit (Bühlmann), on frozen faecal material as described previously.^34^

### Exposure of interest

**Figure 1:**
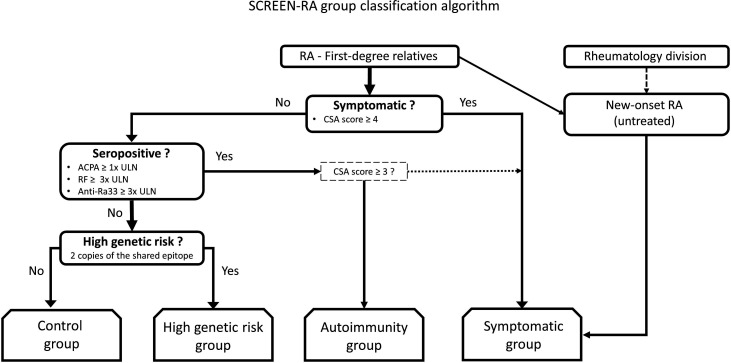
SCREEN-RA group assignation algorithm. Patients with new-onset rheumatoid arthritis (RA) recruited from the rheumatology division are not necessarily RA first-degree relatives. Of note, two cutoffs are possible for clinically suspect arthralgia (CSA) score; in case of one or two missing items, or in case of concomitant autoimmunity, the lower cutoff is applied (CSA score of at least 3). ACPA, anticitrullinated peptide antibodies; CSA, clinically suspect arthralgia, defined using EULAR score; RA, rheumatoid arthritis; RF, rheumatoid factor; ULN, upper limit of normal.

RA-FDRs from the SCREEN-RA cohort^30^ were classified into four preclinical stages (figure 1):

1. Control, that is, healthy asymptomatic RA-FDRs, without clinically significant autoantibody titres (ACPA below the upper limit of normal (ULN), RF <3× ULN, anti-Ra33 <3× ULN)
2. High genetic risk, that is, healthy asymptomatic RA-FDRs with two copies of the shared epitope
3. Autoimmunity, that is, RA-FDRs without articular symptoms but with clinically significant autoimmunity (ACPA titres at least ULN, or RF or anti-Ra33 at least 3× ULN);
4. Symptomatic, that is, RA-FDRs with a CSA score ≥4, using the European Alliance of Associations for Rheumatology questionnaire. When one of the CSA items was missing or if concomitant autoimmunity, a CSA score >3 was used to define clinically suspect symptoms for RA (see criteria in online supplemental table S1).^35^ Finally, this group also includes newly diagnosed RA, that is, RA-FDRs who developed incident RA and a small number of untreated new-onset RA recruited as positive controls, as the number of incident RA cases was insufficient to constitute an independent group.

10.1136/rmdopen-2023-003589.supp1 Supplementary data

This classification is defined at (1) the time of serum sampling for serum-derived variables (except anti-Ra33 titres) and (2) in the 60 days surrounding the date of stool sampling for variables included in the CSA score (maximum score was retained).

As a secondary exposure of interest, we selected from each group only the 20 most pronounced phenotypes (RA diagnosis, then highest CSA scores and autoantibody titres), matching the 20 individuals to controls for sex and age. This subgrouping was pre-planned and used for a parallel project.

### Outcomes

The main outcome was the quantitative abundance of *Prevotellaceae* bacteria in stool samples, expressed as an estimation of the absolute bacterial cell counts per gram of stool (quantitative microbiome profiling, QMP). As a secondary outcome, we examined the percentage of total 16S sequences (relative microbiome profiling, RMP).

Other secondary outcomes also included the abundances of other bacterial families and genera of interest, as well as faecal calprotectin concentration.

### Statistical analyses

#### Population characteristics

Continuous baseline variables were expressed as means with standard deviation (SD). Continuous variables were compared between groups using Kruskal-Wallis test if not normally distributed and analysis of variance (ANOVA) if more than two groups. Categorical variables were described using percentage and compared using χ^2^ test or Fisher’s exact test for small sample sizes. Two-tailed p<0.05 was considered significant. Analyses were conducted using R, V.4.3.0, with package *tableone.*

#### Microbiome

FASTQ files obtained from the MiSeq platform were demultiplexed with the Lotus pipeline (V.1.60).^36^ Then, demultiplexed FASTQ files were filtered and trimmed using the DADA2 pipeline (V.1.16.0) on R (V.4.0.3).^37 38^ Reads were truncated after 230 (forward) and 150 (reverse) nucleotides. Denoising, merging and chimaera removal were performed with default parameters. This generated a set of amplicon sequence variants (ASVs), which were subsequently matched to formatted Silva set ‘SLV_nr99_v138.1’ using the DADA2 built-in assigner.^39^

The output of the DADA2 pipeline was visualised on R with packages *phyloseq* (V.1.32.0) and *ggplot2* (V.3.4.2).^37 40 41^ Sample richness was assessed using the Shannon index. For principal coordinate analysis (PCoA), ASV counts were transformed into proportions, and samples were ordinated using a Bray-Curtis dissimilarity matrix (at the ASV level), before principal component analysis (PCA) plotting. Permutational multivariate analysis of variance (PERMANOVA) was performed on the Bray-Curtis dissimilarity matrix using function *adonis2*() from R package *vegan.*

The QMP matrix was built as described previously.^32^ In brief, samples were downsized to even sampling depth, defined as the ratio between sampling size (16S rRNA gene copy number-corrected sequencing depth) and microbial load (the average total cell count per gram of frozen faecal material). 16S rRNA genome copy numbers were imputed using RasperGade16S,^42^ a new tool that uses a heterogeneous pulsed evolution model for predicting 16S rRNA genome copies (also providing confidence estimates for the predictions). A minimum rarefied read count of <150 was used for QMP analyses. Rarefied ASV counts were converted into numbers of cells per gram.

For enterotyping, observed genus richness was calculated on the genus matrix (downsized to 10 000 reads) using *phyloseq*,^40^ as already reported for previous studies.^34^ Enterotyping (or community typing) based on the Dirichlet multinomial mixture approach was performed in R as described previously.^34 43 44^ It used a combined genus-level abundance RMP matrix including SCREEN-RA samples compiled with 1045 samples originating from the Flemish Gut Flora Project.^45^ The optimal number of Dirichlet components based on the Bayesian information criterion was four. The four clusters were named *Bacteroides1* (Bact1), *Bacteroides2* (Bact2), *Prevotella* (Prev) and *Ruminococcaceae* (Rum) as described previously.^32^

Microbial community composition and differential analysis were conducted using non-parametric tests, such as Wilcoxon rank sum and Kruskal-Wallis. To assess other taxa-specific differences between groups, low abundance ASVs were removed (ie, ASV not present at least 10 times in 5% of the samples). Then ASVs in this filtered dataset were aggregated at the relevant taxonomical level (family or genus level), and sequence counts were compared between groups using R package *Aldex2* accounting for multiple testing and data compositionality (*Aldex2* performs a centred log-ratio transformation on the count data and applies Benjamini-Hochberg correction on p-values). Other p-values were also corrected for multiple testing using the Benjamini-Hochberg method (reported as *p-adj*) when multiple tests were performed on lists of features.

#### Faecal calprotectin

Since non-normally distributed, faecal calprotectin values were compared between groups using Wilcoxon signed rank tests (pairwise, with control group as reference, applying Benjamini-Hochberg correction).

#### Sensitivity analysis

To compare more pronounced phenotypes of the groups, we selected 20 persons with the highest autoantibody titres or arthralgia scores. We compared the differences in median abundances of *Prevotellaceae* using permutation tests with 10 000 permutation samples. For each permutation sample, two groups of 20 individuals were randomly selected from the whole cohort, and differences in median *Prevotellaceae* abundances (proportions) were compared. The one-tailed p-value was estimated by the proportion of permutation samples with a median difference as extreme or more extreme than the median difference between the two pronounced phenotype groups.

## Results

### Population description

**Figure 2:**
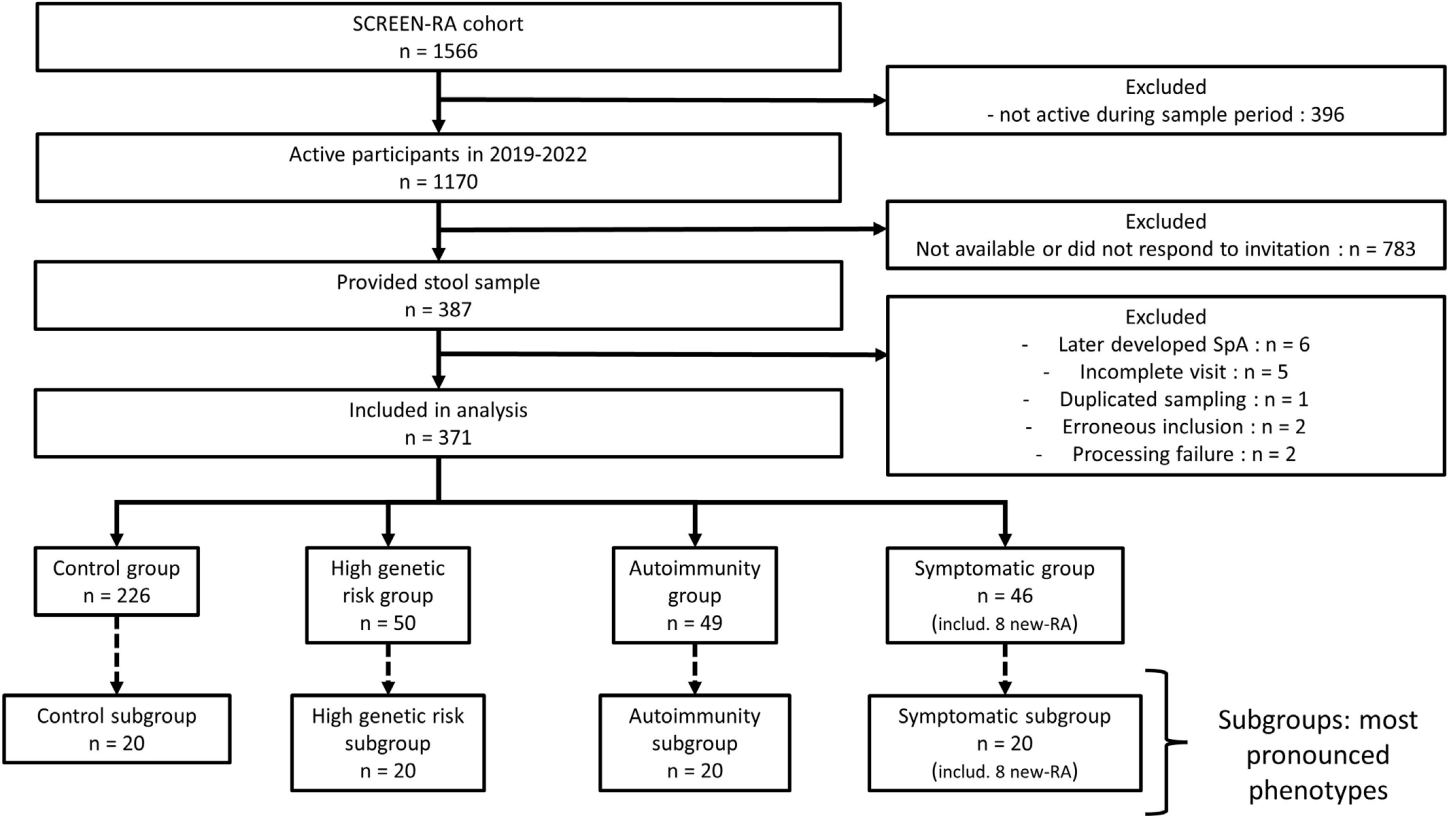
Study recruitment flow chart. New-onset rheumatoid arthritis (RA) includes both first-degree of relatives with RA (RA-FDRs) from SCREEN-RA who developed a new RA at the time of sampling and new-onset RA from the Geneva rheumatology division, which are not necessarily RA-FDRs. SpA, spondyloarthritis; RA, rheumatoid arthritis.

A total of 371 individuals were included in this study (figure 2).

We sampled 226 controls, 50 individuals with high genetic risk, 49 individuals with autoimmunity and 46 symptomatic individuals (including 8 new-onset untreated RA). Baseline characteristics per group are presented in table 1. The subgroups with the most pronounced phenotypes are presented in online supplemental table S2).

**Table 1:** Baseline characteristics of study population, SCREEN-RA

Variable	Control n=226		High genetic risk n=50		Autoimmunity n=49		Symptomatic n=46		p-value
	N % of total in group Otherwise: mean (SD)								
		Miss.		Miss.		Miss.		Miss.	
Female	78%		82%		73%		87%		0.377
Age	52 (14)		53 (12)		55 (16)		54 (12)		0.523
BMI	24 (4)		25 (3)		25 (4)		25 (5)		0.7
Share epitope copie(s) 0 1 2	53% 47% 0%		0% 0% 100%		47% 39% 14%		50% 39% 9%	2%	<0.001
RA autoimmunity	0%		0%		100%		26%		<0.001
ACPA Negative Low High	100% 0% 0%		100% 0% 0%		67% 14% 18%		83% 4% 13%		<0.001
RF Negative Low High	90% 10% 0%		94% 6% 0%		27% 2% 71%		65% 17% 17%		<0.001
Anti-Ra33 Negative Low High	41% 9% 0%	50%	72% 20% 0%	8%	65% 20% 4%	10%	54% 13% 0%	33%	<0.150
Clinically suspect arthralgia (CSA) No Yes	96% 0%	4%	100% 0%		100% 0%		9% 89%	2%	<0.001
CSA score (detail) 1 2 3 4 5 6	64% 23% 10% 0% 0% 0%	4%	80% 16% 4% 0% 0% 0%		82% 18% 0% 0% 0% 0%		4% 4% 9% 61% 11% 9%	2%	<0.001
Antibiotics (past 2 months)	6%		2%		6%		6%		0.710
Probiotics (past month)	10%		8%		8%		9%		0.965
Surgery (past 2 months)	2%		6%		6%		6%		0.162
Travel outside Europe (past month)	2%		2%		2%		0%		0.827

Of note, four patients with new-onset RA included in ‘symptomatic’ group due to their diagnosis, however, did not meet threshold for ‘CSA’ because of either missing data in questionnaires or not having obvious symptoms at the study visit (symptoms can fluctuate and regress between flares).

Note: For technical reasons, anti-Ra33 titres were measured on several previous serum samples using kit Elia anti-Ra33 for IgA, IgG and IgM isotypes (research use only Phadia AB). Hence, the present study imputes the anti-Ra33 serology based on serological measures obtained months to years before the stool sampling of interest, which also explains the higher missing rate when a recent sample with anti-Ra33 dosage was not available.

ACPA, anticitrullinated peptide antibodies; BMI, body mass index; CSA, clinically suspect arthralgia; RA, rheumatoid arthritis; RF, rheumatoid factors.

### Microbiome

**Figure 3:**
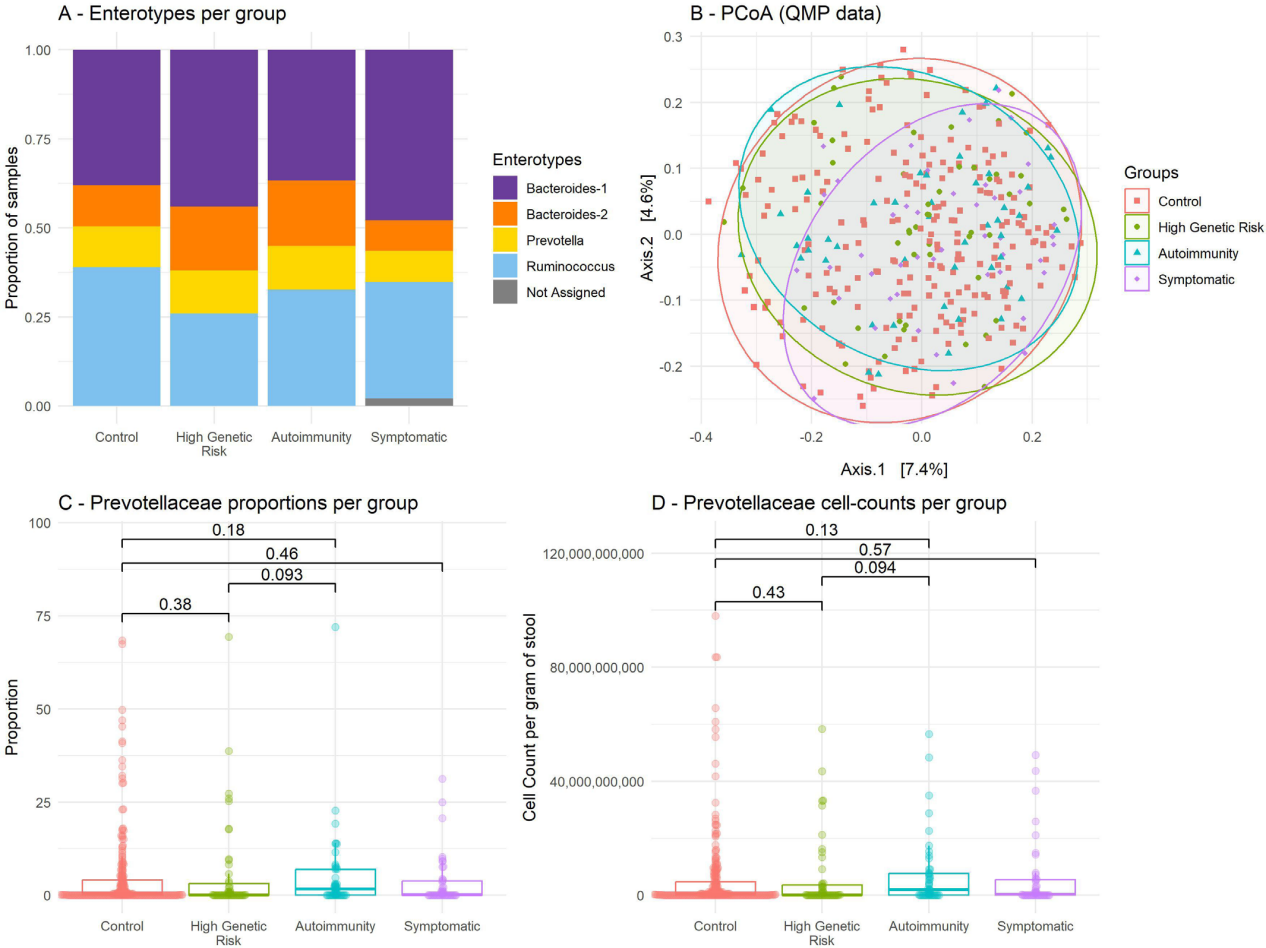
Gut microbiome profiling by group. (A) Enterotype distribution by group; Fisher p=0.64. One sample was not assigned due to low read-count. (B) Principal coordinate analysis performed at the sequence variant level, after quantitative correction (QMP); distance between points reflects their dissimilarity, based on Bray-Curtis’s index. PERMANOVA R2=0.00798; p=0.56. (C) Proportions of *Prevotellaceae* bacteria per group (RMP), boxplots; p-values are Wilcoxon tests. (D) *Prevotellaceae* estimated cell counts per group (QMP), boxplots; p-values are Wilcoxon tests. PCoA, principal coordinate analysis. QMP, quantitative microbiome profiling (provides estimated cell counts). RMP, relative microbiome profiling (provides proportions).

Shannon index, which reflects the number of different bacterial taxa identified in each stool sample (alpha-diversity), did not differ between the groups (online supplemental figure S1). As a gross assessment, each faecal microbiome can be assigned to an enterotype, based on the dominant taxa.^46^ Assigning samples in their respective enterotypes did not reveal significant differences between the groups (Fisher’s exact test p=0.64, figure 3A).

To assess the main outcome (QMP) at the most granular level, it is possible to compare samples pairwise, using Bray-Curtis distance.^47^ This index, ranging from 0 to 1, reflects the ecological difference between two samples, in terms of counts of detected taxa (in our case, the QMP taxonomic counts per gram of stool). Comparing sample compositions to each other using Bray-Curtis index subsequently allows performing a PCoA; on such a figure, the distance between two points increases when their compositional difference increases, as assessed by Bray-Curtis index. We found no group-wise clustering doing a PCoA on the QMP data at the 16S sequence variant level (PERMANOVA, R2=0.00798, p=0.56; figure 3B). Also, using the RMP data (uncorrected bacterial proportions) yielded the same results (PERMANOVA, R2=0.0073, p=0.83). Overall, stool profiling was similar between groups, both when assessed as estimated cell counts and as percentages (see family level, online supplemental figure S2).

More specifically, contrary to our previous report, we found no group differences in *Prevotellaceae* RMP abundance (figure 3C; Kruskal-Wallis p=0.28). Results were similar using the QMP data (online supplemental figure S3, figure 3D; Kruskal-Wallis p=0.29).

To explore differential abundance of other bacterial taxa, as secondary outcomes, we used *Aldex2* tool. It performs centred log-ratio transformations on crude 16S count data and applies Benjamini-Hochberg correction on Kruskal-Wallis p-values, to account for multiple testing. *Aldex2* found no significant differences between groups regarding other bacterial families or genera present in the dataset (online supplemental figure S4). Also, contradicting previous findings,^48^ grouping on shared epitope genotype, we found no association between shared epitope presence and *Prevotellaceae* or *Prevotella* genera (not shown).

#### Microbiome in subgroups

**Figure 4:**
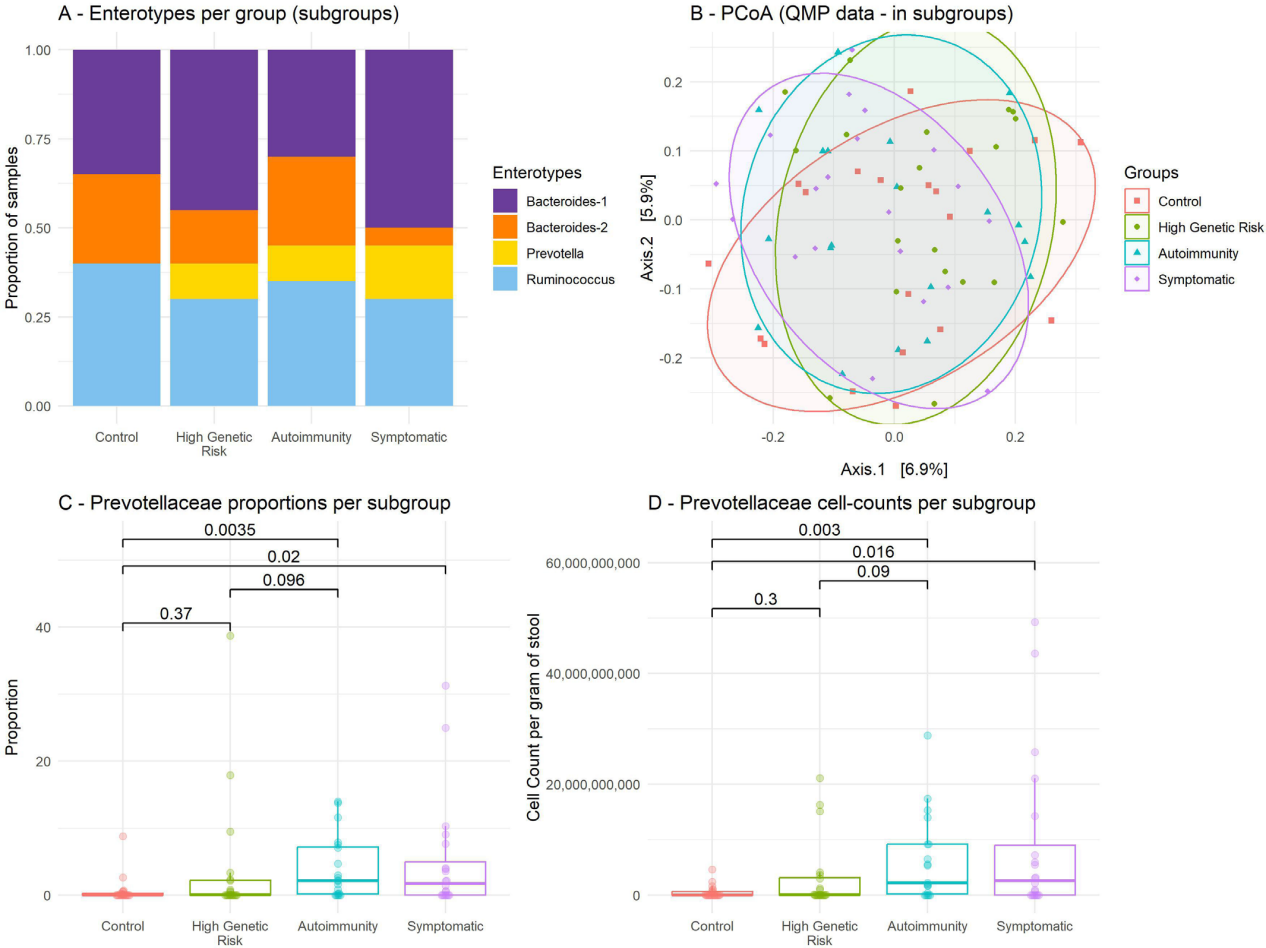
Gut microbiome profiling by subgroup. (A) Enterotype distribution by subgroup; Fisher’s p=0.5473. (B) Principal coordinate analysis performed at the sequence variant level with quantitative data; distance between points reflects their dissimilarity, based on Bray-Curtis’s index. PERMANOVA R2=0.042, p=0.14. (C) Proportions of *Prevotellaceae* bacteria per group, boxplot; p-value is Wilcoxon tests (unadjusted). Adjusted p-values are, from bottom to top, 0.37, 0.13, 0.04 and 0.014, respectively. (D) *Prevotellaceae* estimated cell counts per group, boxplots; p-values are Wilcoxon tests (unadjusted). Adjusted p-values are, from bottom to top, 0.34, 0.13, 0.04 and 0.014, respectively. PCoA, principal coordinate analysis. QMP, quantitative microbiome profiling (provides estimated cell counts). RMP, relative microbiome profiling (provides proportions).

In the sensitivity analysis, we selected the 20 most pronounced phenotypes in each group (for instance, in the symptomatic group: all eigth patients with RA, then highest autoantibody titres or arthralgia scores). Though the increase in *Prevotella* enterotype is still visible for the symptomatic subgroup, it was not significant (figure 4A); also, overall PCoA PERMANOVA remained non-significant (figure 4B). We modestly reproduced published results regarding increased *Prevotellaceae* abundance in autoimmunity and symptomatic groups (online supplemental figure S5), both in RMP (figure 4C) and QMP (figure 4D).

As an alternative to Benjamini-Hochberg method, we reassessed the p-value of these subgroup *Prevotellaceae* differences, by performing a permutation test (10 000 repetitions). Only 5.548% of the permutation samples had a median difference of quantitative abundance (QMP) more extreme than observed in the pronounced phenotype subgroups (if comparing control with autoimmunity), corresponding to a one-sided p-value of 0.054 (0.038 if using RMP data; online supplemental figure S6).

### Faecal calprotectin

**Figure 5:**
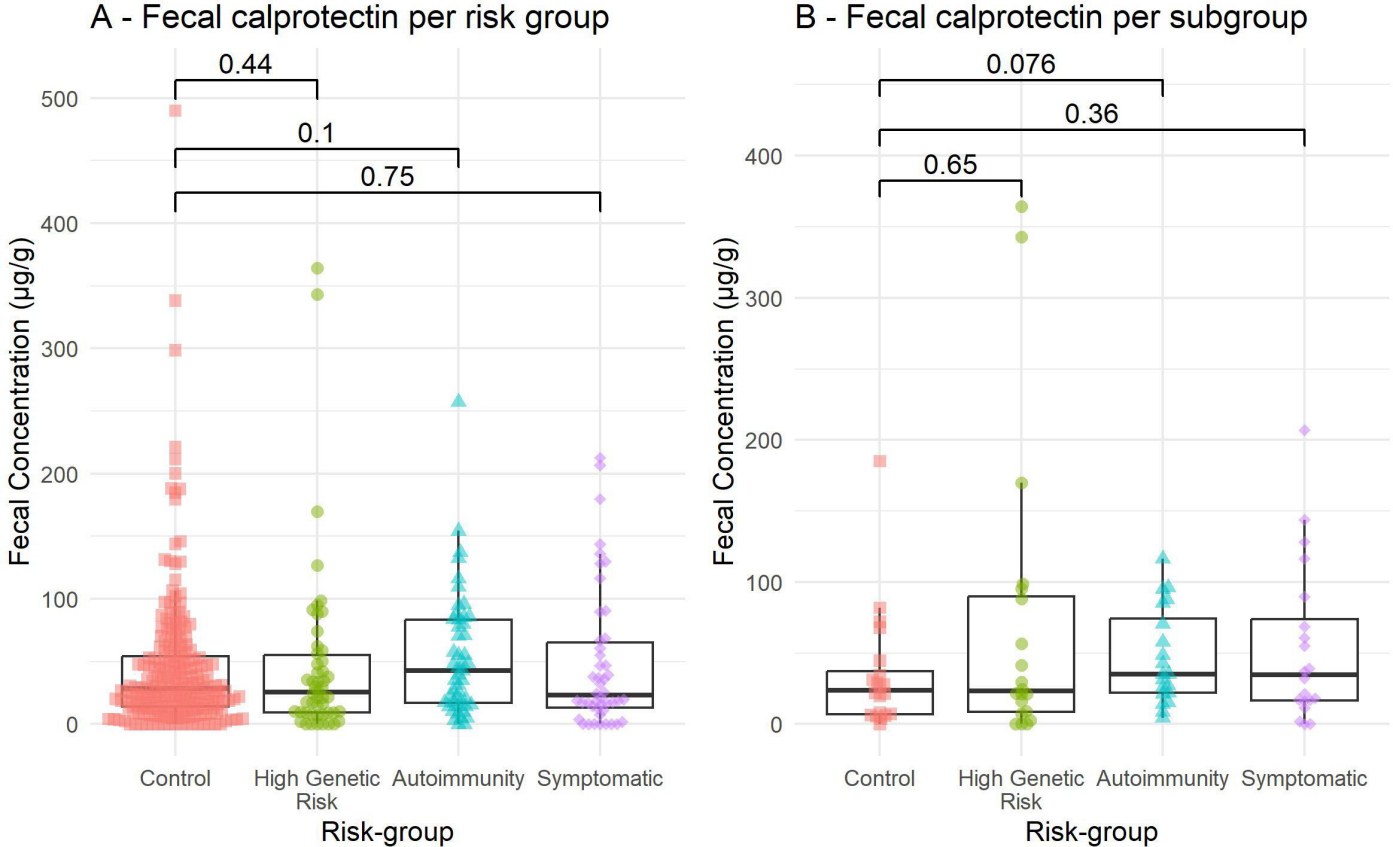
Faecal calprotectin by risk group. Measured with ELISA in fresh frozen stool. (A) In all included stool samples. p-values are Wilcoxon tests. (B) Only in the most pronounced phenotype subgroups. Displayed p-values are Wilcoxon test, non-adjusted. Adjusted p-values for subgroups are, respectively, (from bottom to top) 0.65, 0.55 and 0.23.

Examining a biomarker of mucosal inflammation, we found no overall difference in faecal calprotectin between groups (Kruskal-Wallis p=0.3; figure 5A). When restricting the analysis to the most pronounced subgroups, a trend was noticeable, with a modest increase in the autoimmunity group compared with control group, which disappeared after correction for multiple testing (p=0.076; adjusted p=0.23; figure 5B). Also, *Prevotella* genera were not among the bacteria associated with mildly elevated (>100 ug/g) calprotectin in this dataset, as assessed using *Aldex2* (associated microbes were *Streptococcus* and an unclassified *Clostridia UCG-014*) (data not shown).

## Discussion

In this study, we investigated the association between faecal bacterial composition, faecal calprotectin and different ‘preclinical’ stages of RA. We found no association between ‘dysbiosis’, or specific bacterial taxa, and the preclinical RA grouping. However, when analysing a subgroup of individuals with the most pronounced phenotypes, we retrieved some modest associations in line with the previously reported findings, namely, an increased prevalence of *Prevotellaceae* in later preclinical stages. Also, faecal calprotectin levels did not differ significantly between the groups, which confirms our previous finding on serum biomarkers of intestinal damage as analysed in serum samples from the exact same population.^49^ Still, we noticed a trend for calprotectin elevation in the most pronounced autoimmunity subgroup which became non-significant after p-value correction for multiple testing.

The literature about gut microbiome of RA-FDRs is scarce. A previous study by Rooney *et al* examined the faecal 16S rRNA gene sequences of 25 asymptomatic ACPA-positive individuals and reported distinct feature as compared with 44 unrelated healthy controls (but no difference in *Prevotellaceae*).^50^ Beyond bacterial taxonomy, the faecal bacteriophage community of RA-FDRs was also assessed in a study by Mangelea *et al*, using a smaller sample size (25 individuals divided into 3 groups).^51^ The only cross-sectional study fully comparable with the present manuscript is a previous work from our group, in the same cohort. However, Alpizar *et al* used a simpler exposure (merging autoimmunity and symptomatic groups, without including new onset RA cases), a slightly more stringent control group, a different stool sampling procedure together with a different bioinformatical pipeline (though also 16S based, providing RMP) and an earlier version of the SCREEN-RA database.^12^ These technicalities might partly explain the differences in results. However, as the design is conceptually identical, not being able to reproduce the results while using very similar techniques underlines the tenuousness of such associative findings.

Scher *et al* were the first to report increased proportions of *Prevotellaceae* in untreated new-onset RA patients.^11^ We only recruited eight new-onset RA, precluding any reliable conclusion in this subpopulation; still, at first sight, patients with RA in our study did not have extreme values of *Prevotellaceae* abundances (whether QMP or RMP, not shown). Other microbiome studies in RA have mostly compared treated chronic RA cases with unrelated healthy controls,^13–20 22–24 52 53^ which do not make them exactly comparable to our study setting due to the impact of antirheumatic therapies on microbiome and intestinal health.

### Limitations

The main limitation of this study is related to misclassification of the exposure, in that our classification of at-risk population is based on expert opinion and usual terminology used in the field.^35^ Because our cohort recruits RA-FDRs before they develop RA, we cannot ensure that our ‘higher-risk’ groups are actually comprised of individuals who will develop RA in the future. Of note, since study completion, two individuals newly developed RA, but at the time of stool sampling (~2 years before), they were assigned to the *control* group (seronegative, with no clinically significant symptoms—they later developed ACPA-negative RA). Online supplemental figure S7 illustrates the definitional overlap between groups (PCA, using grouping variables as input).

Our cases and controls are all derived from the same source population of RA-FDRs. By comparing faecal samples from this unique population, we aimed at neutralising confounding by genetic background and maybe overall lifestyle, as well as ensuring clinical applicability of potential findings. However, a drawback of this approach is a more phenotypically homogenous population, making any statistical signal even less prominent, though SCREEN-RA faecal samples were not significantly different from a matched cohort of healthy controls from the Flemish Gut Flora Population (FGFP) with regard to the bacterial families of interest (online supplemental figure S9). Many ‘mild phenotypes’ did not reach the thresholds for CSA or for autoantibody seropositivity and were attributed into our large ‘control’ group (table 1; online supplemental figure S7). Also, the CSA score involves self-reported items and/or nurse’s clinical assessment, which could also lead to exposure misclassification because of limited specificity. Overall, imprecise exposure assessment and non-differential misclassification generate a bias towards the null, which could explain the absence of a clear signal. To address the possibility of a dilution of the effect, we had defined *a priori* a subgroup of participants with more pronounced phenotypes, which did confirm, even though modestly, some of the findings previously reported.^11^

Last but not least, faecal samples are only a proxy of the gut microbiome, and it is unclear to what extent microbes in faeces are informative about the mucosal barrier microenvironment. Microbiome and inflammation on other mucosal sites have also been hypothesised to favour the development of RA autoimmunity, which have not been studied in this analysis. Finally, we have not been able to account for the possible confounding effect of diet, antibiotic treatments or the use of probiotics; however, given the prevalence of these potential confounding factors which was balanced across groups (table 1), we think it is unlikely that they biased our findings.

### Strengths

The main strength of this study is a larger sample size. Our methodology also included for the first time in this population an estimation of faecal bacterial loads, which might be more meaningful than a simple proportion of bacterial taxa, given the high interindividual variability in total faecal biomass.

To avoid confounding by immunosuppressants and antirheumatic treatments, we only enrolled participants without DMARD therapy. The multimodal assessment of serum autoantibodies, faecal inflammatory biomarker and microbiome composition may also provide precious insights into how these parameters covary. In addition, given the long-term follow-up in the SCREEN-RA cohort, the data we generated will be usable retrospectively, if more individuals develop incident RA.

### Perspectives

Even though we confirm the detection of the RA-associated bacterial genera in our cohort of at-risk individuals (online supplemental figure S8), we could not find significant group-wise differential abundances. Also, trying to reproduce Pianta *et al* findings regarding anti-*Prevotella* serum Ig reactivity, Seifert *et al* only retrieved modest results.^54^ Similarly, in a recent work, we were not able to demonstrate significant increases of serum anti-*P. copri* IgG in the context of RA, but we noticed a high variability in reactivity depending on the *P. copri* strain tested.^55^

The latter underlines how using only one bacterium as a biomarker may be too simplistic. Future research should rather explore what these different RA-associated microbes have in common (in terms of gene function, surface antigens, mucus-invading capabilities, etc), while the strain-level variability of *P. copri* should be better accounted for. Alternatively, obtaining gut biopsies from diseased and at-risk individuals would certainly help unravelling host-microbe interactions in the context of RA, but given the inconvenience and ethical issues, this will remain rarely possible. Repeated longitudinal sampling should be considered, to monitor time variation in all these parameters. Still, one could argue that faecal microbiome transfer trial, as recently done in psoriatic arthritis,^56^ might be more a pragmatic way to assess if the gut microbiome impacts RA development. Finally, our results question the transposability mice-microbiome studies to the human disease. As underlined by Walter *et al*, humanised microbiome rodent models often suffer from insufﬁcient rigour in experimental designs and inappropriate statistical analyses, which can result in too optimistic conclusions regarding causality.^57^

### Conclusion

Most microbes previously associated with RA development could be identified in a RA-FDR population. However, the presence of these microbes did not appear to correlate with the known preclinical stages of RA. Yet, in a subgroup analysis of only the most pronounced phenotypes, we noticed a modest signal for increased faecal *Prevotellaceae* abundance, mirroring previous reports. Faecal calprotectin levels did not significantly associate with RA autoimmunity or clinically suspect arthralgia, being normal in most of the enrolled individuals.

10.1136/rmdopen-2023-003589.supp2 Supplementary data

## Data Availability

Data are available upon reasonable request. The raw dataset generated for this study is available upon reasonable request to axel.finckh@hcuge.ch. R-code and processed datasets able to generate the reported figures and tables are publicly available on an institutional deposit at [YARETA link to be updated]. https://doi.org/10.26037/yareta:q2a4hokunzdmhdjrqd7unsnvdm
