# Supplementary material for: Gut microbiome and intestinal inflammation in preclinical stages of rheumatoid arthritis
Source: RMD Open. 2024 Jan 30;10(1):e003589. doi: 10.1136/rmdopen-2023-003589 (PMC10836359; doi:10.1136/rmdopen-2023-003589)
Supplement: Supplementary data [file rmdopen-2023-003589supp001.pdf]

SUPPLEMENTARY MATERIAL

**Table S1: EULAR defined characteristics describing arthralgia at risk for RA**	
**History taking:**	
Joint symptoms of recent onset (duration <1 year)	1
Symptoms located in MCP joints	1
Duration of morning stiffness ≥60 min	1
Most severe symptoms present in the early morning	1
Presence of a first-degree relative with RA	1
**Physical examination:**	
Difficulty with making a fist	1
Positive squeeze test of MCP joints	1
MCP = Metacarpophalangeal. RA = rheumatoid arthritis. Maximum score = 7. In SCREEN-RA, the history-taking items are administered using an online questionnaire.	

Table S2: Baseline characteristics of subgroup study population, SCREEN-RA

Variable	Control n = 20		High Genetic risk n = 20		Autoimmunity n = 20		Symptomatic n = 20		p value
	n % of total in group Otherwise: Mean (SD)								
		Miss.		Miss.		Miss.		Miss.	
Female	95 %		90 %		85 %		95 %		0.632
Age	53 (14)		58 (10)		61 (13)		53 (15)		0.149
BMI	25 (4)		27 (4)		26 (5)		24 (4)		0.260
Share epitope copie(s)									
0	50 %		0 %		45 %		50 %	5 %	<0.001
1	50 %		0 %		40 %		40 %		
2	0 %		100 %		15 %		5 %		
RA autoimmunity	0 %		0 %		100 %		60 %		<0.001
ACPA									
Negative	100 %		100 %		20 %		60 %		<0.001
Low	0 %		0 %		35 %		10 %		
High	0 %		0 %		45 %		30 %		
RF									
Negative	100 %		100 %		60 %		25 %		<0.001
Low	0 %		6 %		5 %		35 %		
High	0 %		0 %		35 %		40 %		
Anti-Ra33									
Negative	50 %		95 %		45 %		45 %		
Low	0 %	50 %	0 %	5 %	25 %	25 %	10 %	45%	0.036
High	0 %		0 %		5 %		0 %		
Clinically Suspect Arthralgia (CSA)									
No	100 %		100 %		100 %		20 %	5 %	<0.001
Yes	0 %		0 %		0 %		75 %		
CSA score (detail)									
1	100 %		100 %		80 %		10 %		
2	0 %		0 %		20 %		10 %		
3	0 %		0 %		0 %		5 %	5 %	<0.001
4	0 %		0 %		0 %		55 %		
5	0 %		0 %		0 %		10 %		
6	0 %		0 %		0 %		5 %		
Antibiotics (past 2 months)	5 %		5 %		10 %		5 %		0.901
Probiotics (past month)	5 %		15 %		5 %		10 %		0.591
Surgery (past 2 months)	0 %		10 %		5 %		5 %		0.528
Travel outside Europe (past month)	5 %		0 %		5 %		0 %		0.583

SD = standard Deviation. BMI = Body Mass Index. RA = Rheumatoid Arthritis. ACPA = Anti-citrullinated Peptide Antibodies. RF = Rheumatoid Factors. CSA = Clinically Suspect Arthralgia.
Of note, 4 patients with new-onset RA included in "symptomatic" group due to their diagnosis, however, did not meet threshold for "CSA" because of either missing data in questionnaires or not having obvious symptoms at the study visit (symptoms can fluctuate and regress between flares).
Note: For technical reasons, anti-Ra33 titers were measured on several previous serum samples using kit: ELIA anti-RA33 for IgA, IgG and IgM isotypes (research use only, Phadia AB). Hence, the present study imputes the anti-Ra33 serology based on serological measures obtained months to years before the stool sampling of interest ; which also explains the higher missing rate when a recent sample with anti-Ra33 dosage was not available.

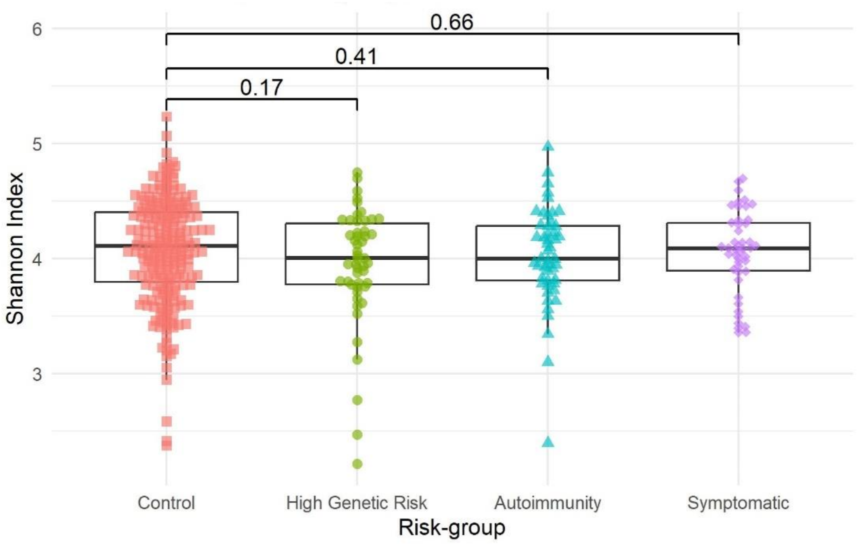

Figure S1: Shannon Index per risk group. The Shannon index reflects the diversity of the microbiome. The higher the score, the higher the number of different amplicon sequence variants (taxonomic units) found in the sample (after DADA2 pipeline processing). P-values from Wilcoxon tests.

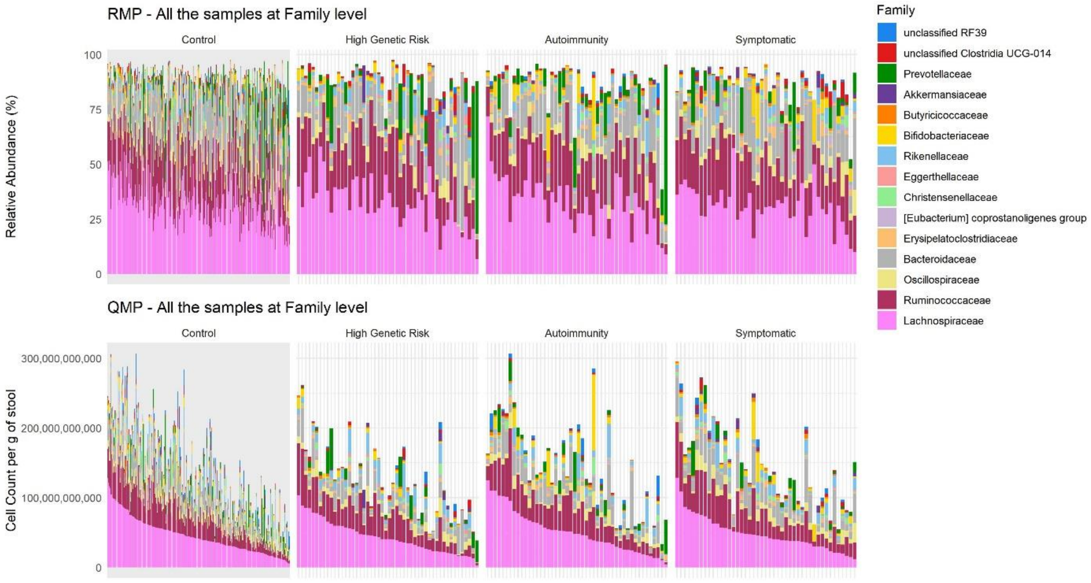

Figure S2: Microbiome profiling at the family taxonomic level, by group. RMP = Relative Metagenomic Profiling (provides proportions). **QMP** = Quantitative Metagenomic Profiling (provides estimated cell counts). Each vertical bar is a sample, colored for the most frequent bacterial families found in the data set. Sample are ordered based on the most prevalent family, using the QMP profiling as reference. Each RMP sample sits above its corresponding QMP profile. To keep the figure readable only the top 15 most represented taxa are represented (on a total of 163, most of which have a negligible prevalence).

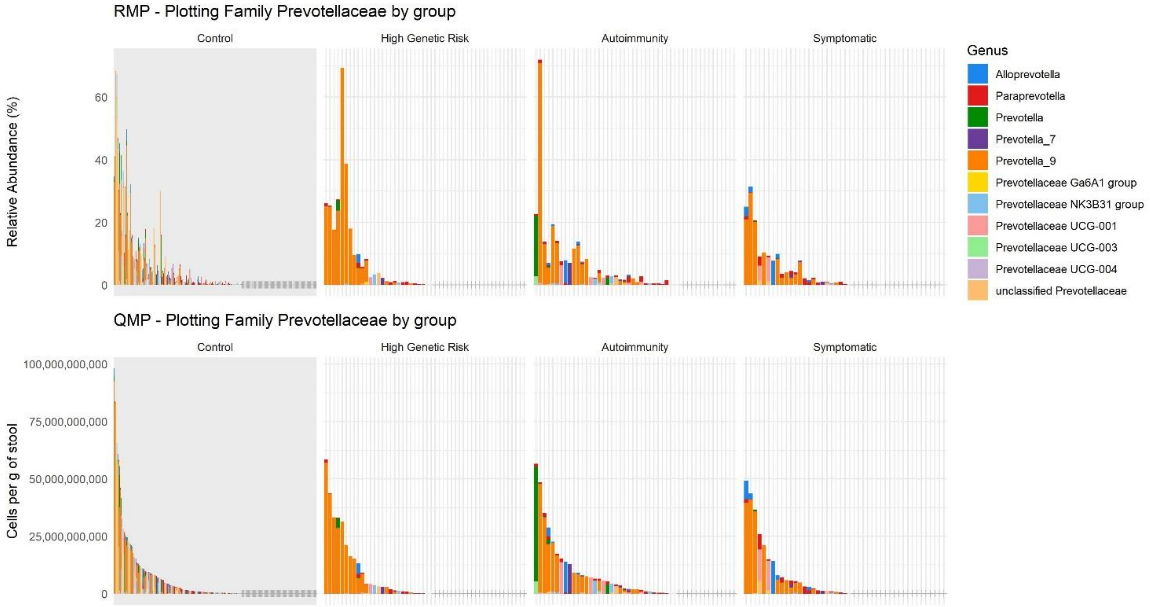

Figure S3: ***Prevotellaceae*** **abundance by sample, by group.** RMP = Relative Metagenomic Profiling (provides proportions). QMP = Quantitative Metagenomic Profiling (provides estimated cell counts). Each bar represents one sample, colored by the different genera identified in the *Prevotellaceae* family. “Prevotella_9” is the annotation for what is mostly *P copri*. Overall Kruskal-Wallis p = 0.2879 (on relative abundances).

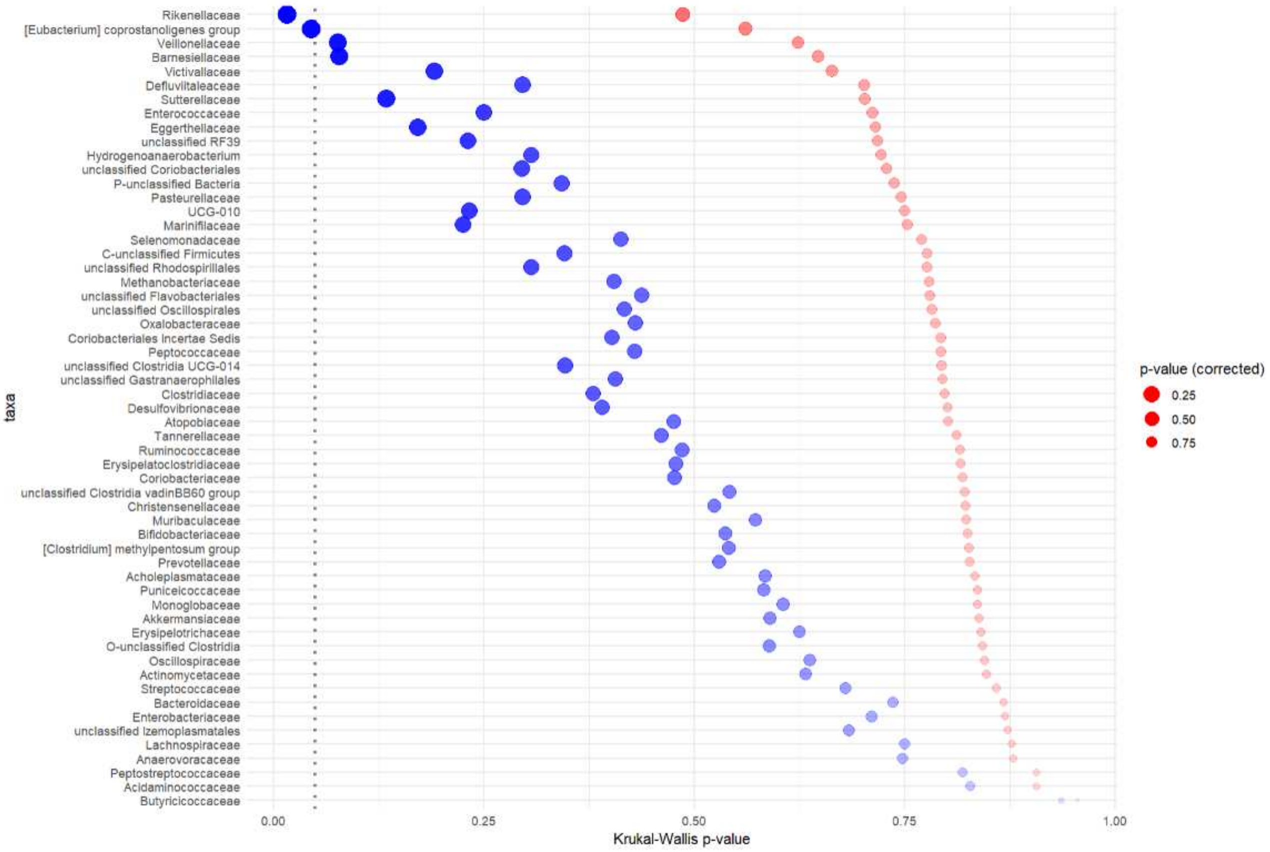

Figure S4: differential abundance analysis performed with Aldex2 (Family level). In this analysis, rare taxa were removed from the dataset, and sequence counts were aggregated at the Family level. Then, *Aldex2* performed a centered-log-ratio transformation (which accounts for data compositionality), and performed serial Kruskal-Walis tests between groups, for every bacterial Family. The blue points represent the raw p-values, the red points are the p-values corrected by Benjamini-Hochberg procedure. Vertical dotted line is the significance threshold (0.05). Point size is inversely related to p-value.

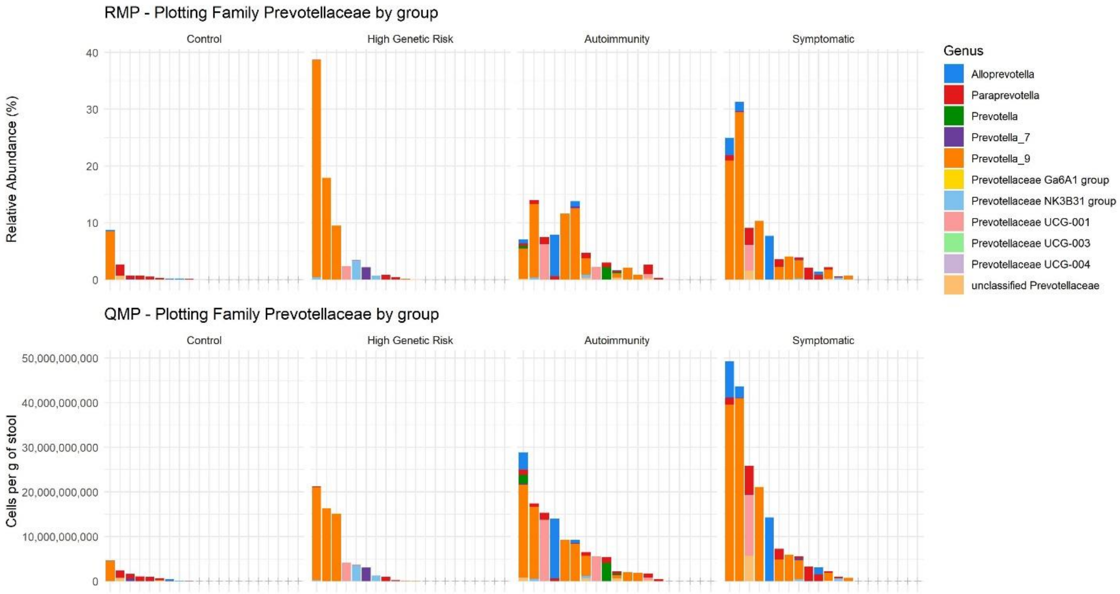

Figure S5: *Prevotellaceae* abundance by sample, in most pronounced subgroups. RMP = Relative Metagenomic Profiling (provides proportions). QMP = Quantitative Metagenomic Profiling (provides estimated cell counts). Each bar represents one sample, colored by the different genera identified in the *Prevotellaceae* family. “Prevotella_9” is the annotation for what is mostly *P copri*. Overall Kruskal-Wallis p = 0.02074 (on relative abundances).

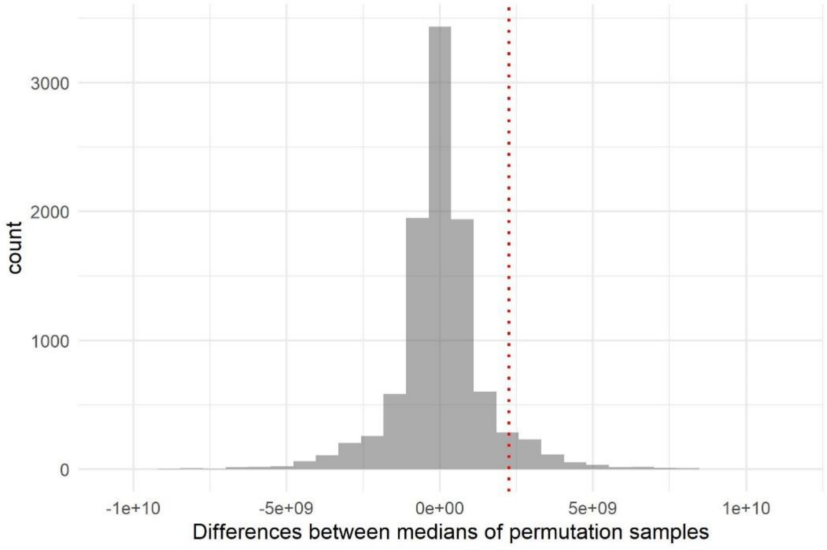

Figure S6: Permutation test on subgroups analysis. This figure assesses the probability that the results reported in Figure S5 are obtained by chance. To do so, we randomly selected two groups of 20 individuals in the cohort and compared their median *Prevotellaceae* quantitative abundances (QMP), computing the difference. This figure represents the median differences obtained after 10’000 repetitions. The vertical redline is the “real” difference between median *Prevotellaceae* abundance in control subgroup versus autoimmunity subgroup. Only 5.548 % percents of random sets fell on the right of the red line, meaning that the one-sided p-value for our finding is 0.054 (0.375 if using the RMP data).

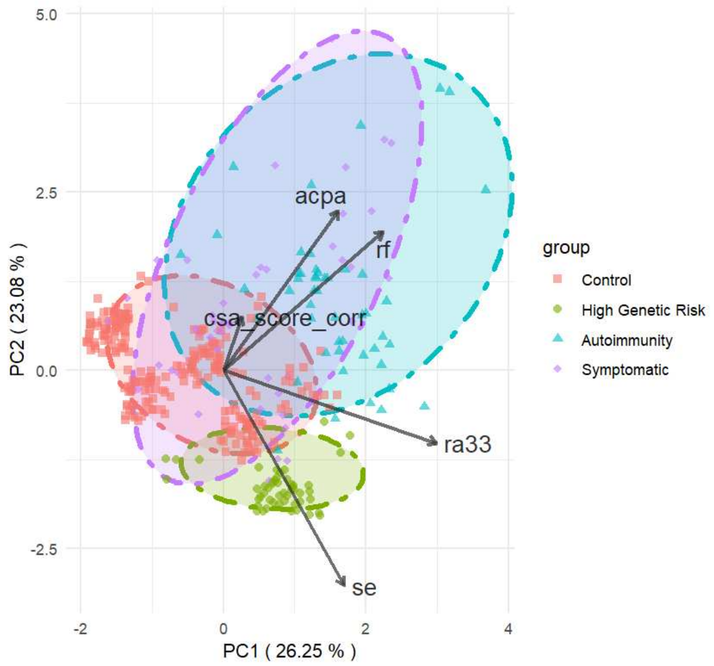

Figure S7: Principal Component Analysis (PCA) of grouping variables. This figure aims at illustrating how phenotypically close the groups of interest are. Hence, a PCA was made using the group variables only. This reveals a substantial overlap, which means that individual grouped as "symptomatic" only slightly differ from "autoimmunity", "High genetic risk" and "control" individuals. *Nota bene*: for proper display a small amount of random noise was added to point coordinates (hence the noticeable clusters of control points are in realty superimposed). ACPA = Anti-citrullinated proteins antibodies. RF = Rheumatoid Factors. CSA_score_corr : Clinically Suspect Arthralgia score (corrected to keep highest value in the 60 days around stool sampling). Ra33 = anti-ra33 antibodies. SE = Share Epitope.

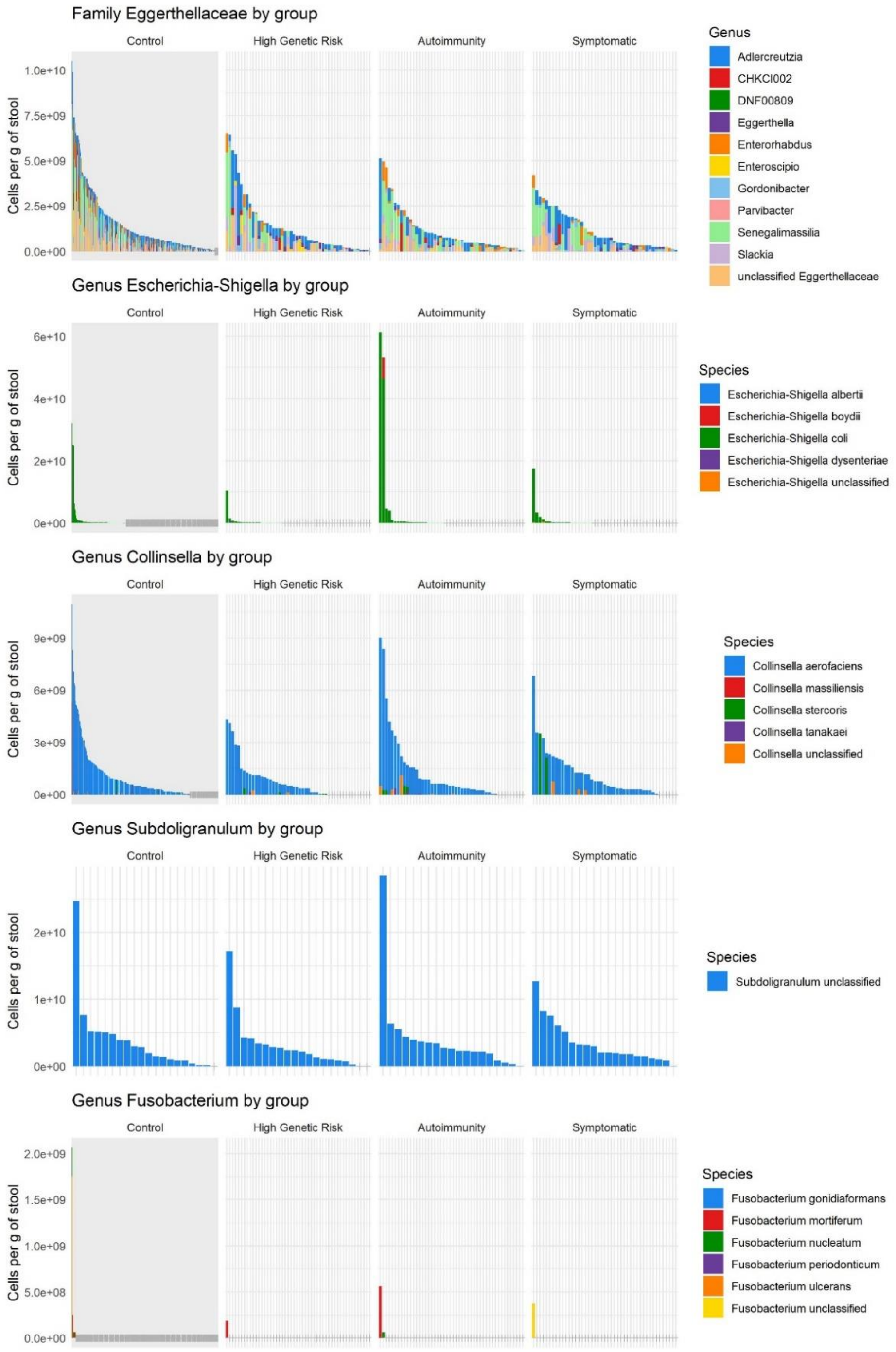

Figure S8: Bacterial loads of RA-associated taxa. Except for *Fusobacterium*, which was only detectable in a dozen of individuals, other bacteria recently associated with RA were commonly detected in our population of interest; however, we did not notice significant differences between groups.

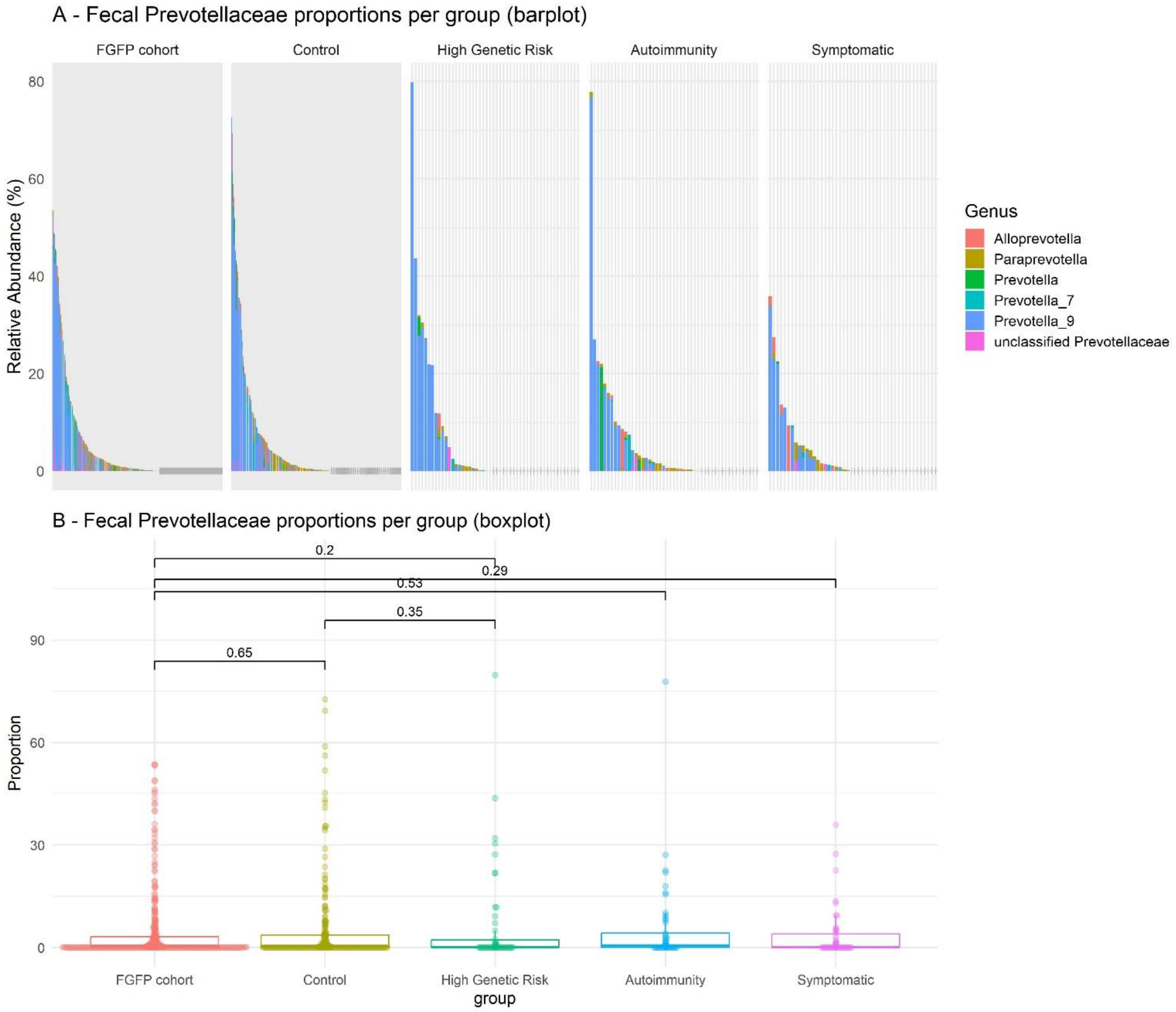

Figure S9: Relative abundance of *Prevotellaceae* in SCREEN-RA groups compared to FGFP healthy controls. On reviewer request, the SCREEN-RA dataset was merged with 353 healthy samples from the Flemish Gut Flora Project (FGFP) (restricting to bacterial taxa found in both sets), matched for sex, age and BMI. **A** - This plot displays the proportions of *Prevotellaceae* – there were no significant differences between the SCREEN-RA groups and the FGFP cohort (Wilcoxon tests). **B** – Same data shown as boxplots (with Wilcoxon p-values). Considering other genera, using Aldex2 algorithm, we found in a very minor though significant magnitude more *Monoglobaceae, Eggerthellaceae, Enterococcaceae, Oscillospirales* in the SCREEN-RA cohort, and less *Muribaculaceae, Rhodospirillales* in the SCREEN-RA cohort than in the FGFP population. Such small differences most likely result from the different lifestyles of the two populations, or from technical biases in this post-hoc analysis (i.e. batch effect). Overall, both cohorts are comparable and mostly overlap indistinctly on a Principal Coordinate Analysis (PCoA) (not shown). After performing the PERMANOVA test between the two cohorts on a species-level Bray-Curtis distance matrix, it was found that 0.8% of the microbial community variation can be explained by country/geographical location (PERMANOVA test, n=total, R2=0.008, p-value <0.05).
